# Mechanism of the blood pressure-lowering effect of sodium-glucose cotransporter 2 inhibitors in obese patients with type 2 diabetes

**DOI:** 10.1186/s40360-017-0125-x

**Published:** 2017-04-10

**Authors:** Shin Kawasoe, Yukiko Maruguchi, Shoko Kajiya, Hitoshi Uenomachi, Masaaki Miyata, Mariko Kawasoe, Takuro Kubozono, Mitsuru Ohishi

**Affiliations:** 1 Department of Cardiovascular Medicine and Hypertension, Graduate School of Medical and Dental Sciences, Kagoshima University, 8-35-1 Sakuragaoka, Kagoshima, 890-8520 Japan; 2 Uenomachi-Kajiya Clinic, Kagoshima, Japan

**Keywords:** Blood pressure, Natriuresis, Osmotic diuresis, SGLT2 inhibitor, Type 2 diabetes

## Abstract

**Background:**

Sodium-glucose cotransporter 2 (SGLT2) inhibitors are reported to have BP-lowering effect in addition to blood glucose-lowering effect, however, its mechanism is still unknown. This study aimed to investigate the mechanism of blood pressure (BP) lowering effects of SGLT2 inhibitors using 24-h urinary collection in obese type 2 diabetes patients.

**Methods:**

Twenty patients with type 2 diabetes (age 48.2 ± 10.7 years, BMI 33.0 ± 4.9 kg/m^2^) were enrolled. Urine volume, 24-h urinary glucose and sodium excretion, and BP at baseline and 2 weeks and 6 months after administration were measured. Body weight, glycosylated hemoglobin, and BP were evaluated before and 1, 3, and 6 months after SGLT2 inhibitor administration. We evaluated the changes in urine volume and urinary excretion of glucose and sodium as well as correlations among urine volume and urinary sodium glucose excretion at 2 weeks and 6 months after administration of the SGLT2 inhibitors. Furthermore, we investigated the correlations between changes in BP and urinary excretion of sodium and glucose at the same time.

**Results:**

Two weeks after administration, systolic BP (SBP) significantly decreased (128.5 ± 11.0 to 123.2 ± 9.8 mmHg, *P* = 0.0314), but diastolic BP (DBP) did not (74.4 ± 10.4 to 73.4 ± 8.5 mmHg, *P* = 0.5821). The decreased SBP significantly correlated with increased urinary glucose excretion (R = −0.62, *P* = 0.0073), but not increased urinary sodium excretion. At 6 months, SBP (118.6 ± 11.0 mmHg, *P* = 0.0041) and DBP (68.4 mmHg, *P* = 0.0363) significantly decreased. The decreased SBP significantly correlated with increased urinary sodium excretion (R = −0.60, *P* = 0.0014), but not increased urinary glucose excretion.

**Conclusions:**

SGLT2 inhibitors significantly decreased SBP after 1 month and DBP after 6 months in obese patients with type 2 diabetes. The main mechanism of the BP-lowering effect may be plasma volume reduction by osmotic diuresis at 2 weeks and by natriuresis at 6 months after SGLT2 inhibitor administration.

## Background

Cardiovascular disease is the major cause of morbidity and mortality in patients with type 2 diabetes. Many patients with type 2 diabetes have various cardiovascular risk factors, and a multifactorial approach to address cardiovascular risk, including controlling glycemia, blood pressure (BP), lipid profile, and body weight, is recommended in these patients. Hypertension affects approximately two-thirds of patients with diabetes, which synergistically adds to the cardiovascular risks and mortality [1, 2]. Lowering BP has been shown to reduce cardiovascular events more than controlling glucose levels in patients with diabetes [3].

Sodium-glucose cotransporter 2 (SGLT2) inhibitors are antidiabetic agents that correct hyperglycemia by promoting urinary excretion through the inhibition of glucose reabsorption in proximal tubules irrespective of the patient’s insulin secretory capacity or insulin resistance [4]. Because this underlying pharmacological mechanism is novel and clearly different from that of other antidiabetic drugs, SGLT2 inhibitors may present a new treatment option for patients with type 2 diabetes.

Several reports have suggested that in addition to its blood glucose-lowering effects, SGLT2 inhibitors exert various other effects such as lowering BP [5, 6], improving the serum lipid profile [7], decreasing body weight [6, 8], and improving insulin resistance [9]. Recently, in the EMPA-REG OUTCOME trial, the SGLT2 inhibitor empagliflozin significantly reduced the rate of primary composite cardiovascular outcome and death from any cause in patients with type 2 diabetes at high cardiovascular risk [10]. This was the first study reporting that an antidiabetic agent reduced mortality in patients with type 2 diabetes. Moreover, the additional use of SGLT2 inhibitors significantly reduced the onset and worsening of nephropathy in the subgroup analysis of the EMPA-REG OUTCOME trial [11]. There are several findings showing that SGLT2 inhibitors decrease BP [12, 13]. However, the mechanism of the BP-lowering effects of SGLT2 inhibitors has not been fully elucidated. For effective use of SGLT2 inhibitors, understanding this mechanism is important. Therefore, we investigated the mechanism of the BP-lowering effects of SGLT2 inhibitors in Japanese patients with type 2 diabetes using 24-h urinary collection.

## Methods

### Study Population

We investigated the outpatients who attended the Uenomachi-Kajiya Clinic (Kagoshima, Japan) from March to August 2014. The eligible patients were men and women with type 2 diabetes over 20 years of age who had inadequate body weight control defined as BMI ≥ 30 kg/m^2^ and poor glycemic control defined as glycosylated hemoglobin (HbA1c) > 6.5% and who were treated with diet/exercise therapy alone or in combination with a stable dose of oral antidiabetic agents (biguanide, sulfonylurea, glinide, α-glucosidase inhibitor, thiazolidinedione, and dipeptidylpeptidase-4 inhibitor) and/or insulin for at least 4 weeks before study entry. Patients with type 1 diabetes and those with a history of ischemic heart disease or stroke were excluded. The study patients were allocated to additional use of SGLT2 inhibitors according to their clinical history and need.

This study conformed to the Declaration of Helsinki and was approved by the Institutional Ethics Committee at Uenomachi-Kajiya Clinic. All subjects gave informed consent before enrollment in the study.

### Data Collection at Baseline and Intervention

Demographic information, medical history, and drug use data were routinely collected. The blood samples were obtained and routine biochemical parameters were measured according to standard techniques. We evaluated body weight, HbA1c, and BP at baseline. Further, we measured urine volume and urinary excretion of glucose and sodium using 24-h urinary collection before administration of SGLT2 inhibitors. HbA1c levels were determined using ADAMS A1c, HA-8180 glycohemoglobin analyzer (ARKRAY, Koka, Japan). The urinary sodium concentrations were measured using STAX-3 electrolyte Analyzer (Techno Medica, Yokohama, Japan) and the urinary glucose concentrations were analyzed using GA-1160 (ARKRAY).

The SGLT2 inhibitors were orally administered to the study patients before breakfast once daily. We did not determine the specific kinds of SGLT2 inhibitors that should be used in this study and started with the usual dosage of these drugs. All patients were maintained on diet therapy to ensure a consistent caloric intake during the entire study period. The patients continued exercise therapy if it was prescribed before study entry to ensure a consistent exercise regimen. Patients were permitted to remain on any lipid-lowering and antihypertensive agents that they had received before study entry. There were no patients who were prescribed diuretics.

### Follow-up

The patients were followed up at an outpatient department and monitored any adverse events resulting from taking SGLT2 inhibitors. They were permitted to decrease the dosage of antidiabetic agents except for the administered SGLT2 inhibitors or antihypertensive agents, if necessary. We measured urine volume and urinary excretion of glucose and sodium using 24-h urinary collection at 2 weeks and 6 months after administration of SGLT2 inhibitors. Furthermore, we evaluated body weight, HbA1c levels and BP 2 weeks and 1, 3, and 6 months after administration of the SGLT2 inhibitors.

### Statistical Analysis

Because SGLT2 inhibitors have similar chemical structures, we analyzed the subjects taking all kinds of SGLT2 inhibitors together. We evaluated the changes in urine volume and urinary excretion of glucose and sodium as well as correlations among urine volume and urinary sodium glucose excretion at 2 weeks and 6 months after administration of the SGLT2 inhibitors. Furthermore, we investigated the correlations between changes in BP and urinary excretion of sodium and glucose at the same time. We compared body weight, BMI, HbA1c levels, and BP at baseline with those at 1, 3, and 6 months after administration of the SGLT2 inhibitors. Finally, we compared BP at baseline with that at 6 months after administration of the SGLT2 inhibitors in patients who were or were not taking baseline antihypertensive drugs. Continuous variables, including age, body weight, BMI, BP, and laboratory and urinary data are expressed as mean ± SD, and categorical variables, including sex and medication history are given as number of subjects and proportions (percentages). Comparisons of the variables determined before and after the administration of SGLT2 inhibitors were analyzed by a paired Student’s *t*-test. Pearson’s simple correlation coefficients were performed to determine the correlations among the changes in BP, body weight, and urinary data.

All statistical analyses were performed with JMP pro version 11 (SAS Institute Inc., Cary, NC, USA) for Windows, and statistical significance was considered to be *P* < 0.05.

## Results

### Baseline Characteristics

The clinical characteristics of patients in the present study are summarized in Table 1. The mean age of the 20 patients was 48.2 ± 10.7 years old; 10 patients (50.0%) were men. The mean body weight, BMI, and HbA1c level were 91.7 ± 22.3 kg, 33.0 ± 4.9 kg/m^2^, and 9.2 ± 1.4%, respectively. The mean systolic BP (SBP) was 128.5 ± 11.0 mmHg and the mean diastolic BP (DBP) was 74.4 ± 10.4 mmHg. The mean serum creatinine and blood urea nitrogen levels were 0.63 ± 0.16 mg/dL and 13.0 ± 3.9 mg/dL, respectively. Nineteen patients (95%) took oral antidiabetic medications other than SGLT2 inhibitors, and 10 patients (50%) were being treated with insulin injection therapy. Eleven patients (55%) were prescribed antihypertensive medications.

**Table 1:** Basic characteristics of the patients

Age (years)	48.2 ± 10.7
Gender (male/female)	10/10
Body weight (kg)	91.7 ± 22.3
Body mass index (kg/m2)	33.0 ± 4.9
Systolic blood pressure (mmHg)	128.5 ± 11.0
Diastolic blood pressure (mmHg)	74.4 ± 10.4
BUN (mg/dL)	13.0 ± 3.9
Creatinine (mg/dL)	0.63 ± 0.16
HbA1c (%)	9.2 ± 1.4
Medication	
Anti-hypertensive agents (%)	11 (55%)
Lipid-regulating agents (%)	10 (50%)
Oral anti-diabetic agents (%)	19 (95%)
Insulin (%)	10 (50%)

*BUN* blood urea nitrogen; *HbA1c* glycosylated hemoglobin

### Changes in HbA1c, body weight, and BP after administration of SGLT2 inhibitors

**Fig. 1:**
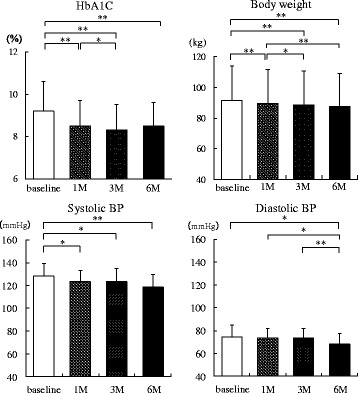
Changes in glycosylated hemoglobin, body weight, and BP up to 6 months after administration of sodium-glucose cotransporter 2 inhibitors (*n* = 20). HbA1c, glycosylated hemoglobin; BP, blood pressure; M, months. *, *P* < 0.05; **, *P* < 0.01

The SGLT2 inhibitors in the present study were ipragliflozin in seven patients, dapagliflozin in six patients, tofogliflozin in four patients, and luseogliflozin in three patients. Figure 1 shows the changes in HbA1c level, body weight, and BP up to 6 months after administration of SGLT2 inhibitors. The HbA1c level began to decrease significantly at 1 month (8.5 ± 1.2%, *P* = 0.0014) and was still significantly decreased at 3 months (8.3 ± 1.2%, *P* < 0.0001). Although the HbA1c level at 6 months (8.5 ± 1.1%) was slightly increased compared with that at 3 months, it remained significantly decreased compared with the pre-administration level (*P* < 0.0001). Body weight was significantly decreased at 1 month (89.5 ± 22.1 kg, *P* < 0.0001), 3 months (88.4 ± 22.3 kg, *P* < 0.0001), and 6 months (87.6 ± 21.4 kg, *P* < 0.0001) compared with baseline. SBP decreased significantly at 1 month (123.2 ± 9.8 mmHg, *P* = 0.0314), 3 months (123.2 ± 11.7 mmHg, *P* = 0.0311), and 6 months (118.6 ± 11.0 mmHg, *P* = 0.0041) compared with pre-administration BP. DBP at 6 months (68.4 ± 9.3 mmHg) was significantly decreased compared with that at baseline (74.4 ± 10.4 mmHg, *P* = 0.0363), 1 month (73.4 ± 8.5 mmHg, *P* = 0.0456), and 3 months (73.4 ± 8.6 mmHg, *P* = 0.0101).

### Changes in Urinary Data After Administration of SGLT2 Inhibitors

**Fig. 2:**
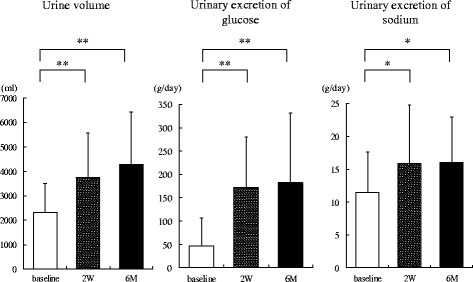
Changes in 24-h urinary collection data up to 6 months after administration of sodium-glucose cotransporter 2 inhibitors (*n* = 20). W, weeks; M, months; *, *P* < 0.05; **, *P* < 0.01

Figure 2 shows the changes in urinary data up to 6 months after administration of SGLT2 inhibitors. The urine volume was significantly increased at 2 weeks (3752 ± 1815 mL/day, *P* < 0.0001) and 6 months (4294 ± 2151 mL/day, *P* < 0.0001) after administration of SGLT2 inhibitors compared with baseline (2309 ± 1186 mL/day). The urinary excretion of glucose at 2 weeks (172.5 ± 107.7 g/day, *P* < 0.0001) and 6 months (182.2 ± 150.3 g/day, *P* < 0.0001) was significantly increased compared with baseline (46.2 ± 60.9 g/day). Furthermore, the urinary excretion of sodium at 2 weeks (15.9 ± 8.9 g/day, *P* = 0.0266) and 6 months (16.1 g/day, *P* = 0.0206) was also significantly increased compared with baseline (11.5 ± 6.1 g/day).

### Relationship Between Changes in Urine Volume and Urinary Excretion of Sodium and Glucose After Administration of SGLT2 Inhibitors

**Fig. 3:**
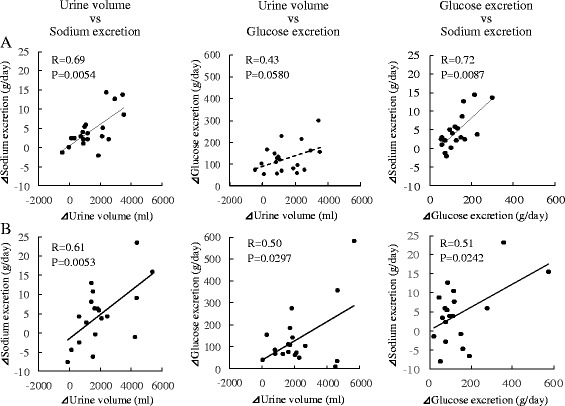
Relationship between changes in urine volume and urinary excretion of sodium and glucose 2 weeks (**a**) and 6 months (**b**) after administration of sodium-glucose cotransporter 2 inhibitors (*n* = 20)

Figure 3 demonstrates the correlations between changes in urine volume and urinary excretion of sodium and glucose 2 weeks (Fig. 3a) and 6 months (Fig. 3b) after administration of SGLT2 inhibitors. At 2 weeks, there were significant correlations between the changes in urine volume and sodium excretion (R = 0.69, *P* = 0.0054) and between the changes in glucose excretion and sodium excretion (R = 0.72, *P* = 0.0087). The change in urine volume tended to correlate with the change in glucose excretion, but this did not reach statistical significance (R = 0.43, *P* = 0.0580). At 6 months, there were significant correlations between the changes in urine volume and sodium excretion (R = 0.61, *P* = 0.0053), between the changes in urine volume and glucose excretion (R = 0.50, *P* = 0.0297), and between the changes in glucose excretion and sodium excretion (R = 0.51, *P* = 0.0242).

### Relationship Between Changes in BP and Urinary Excretion of Sodium and Glucose After Administration of SGLT2 Inhibitors

**Fig. 4:**
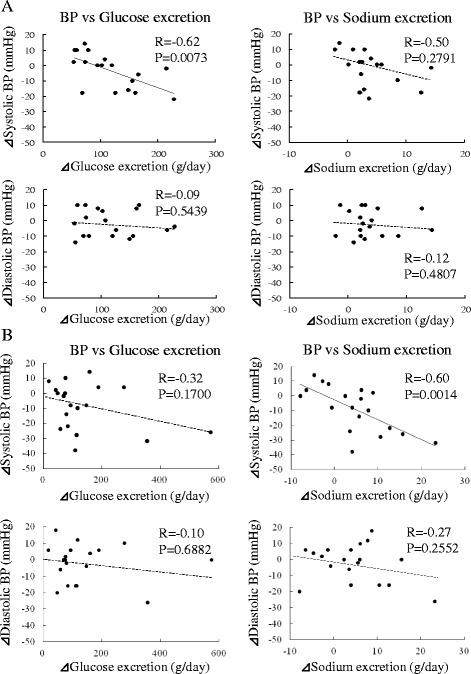
Relationship between changes in BP and urinary excretion of sodium and glucose 2 weeks (**a**) and 6 months (**b**) after administration of sodium-glucose cotransporter 2 inhibitors (*n* = 18). BP, blood pressure

Figure 4 demonstrates the correlations between changes in BP and urinary excretion of sodium and glucose 2 weeks (Fig. 4a) and 6 months (Fig. 4b) after administration of SGLT2 inhibitors. At 2 weeks, the decrease in SBP was significantly correlated with the increase in urinary glucose excretion (R = −0.62, *P* = 0.0073), but not urinary sodium excretion. In contrast, at 6 months, the decrease in SBP was significantly correlated with the increase in urinary sodium excretion (R = −0.60, *P* = 0.0014), but not urinary glucose excretion. There were no significant correlations between the decrease in DBP and the increase in urinary glucose or sodium excretion at 2 weeks and 6 months.

### Changes in BP in Patients Taking or Not Taking Antihypertensive Medications After Administration of SGLT2 Inhibitors

**Fig. 5:**
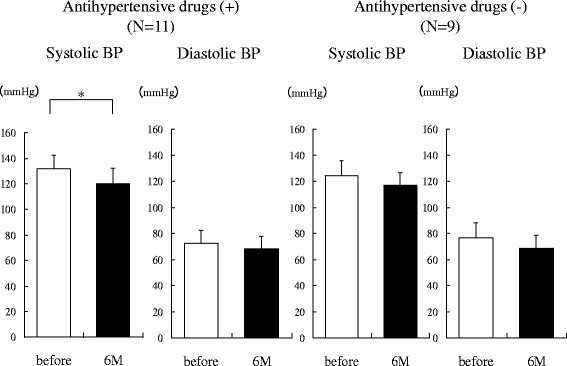
Changes of blood pressure with or without antihypertensive drugs 6 months after administration of sodium-glucose cotransporter 2 inhibitors (*N* = 20). BP, blood pressure; M, months; *, *P* < 0.05

Figure 5 shows the BP before and 6 months after administration of SGLT2 inhibitors in patients with or without antihypertensive medications. In patients taking antihypertensive agents (*n* = 11), SBP at 6 months was significantly decreased compared with baseline (131.8 ± 10.2 vs. 120.0 ± 12.1 mmHg, respectively, *P* = 0.0191), but the DBP at 6 months did not show a significant decrease (72.4 ± 9.9 vs. 68.2 ± 9.4 mmHg). In patients not taking antihypertensive medications (*n* = 9), both SBP (124.4 ± 11.0 vs. 116.9 ± 9.9 mmHg) and DBP (76.9 ± 11.0 mmHg vs. 68.7 ± 9.7 mmHg) tended to decrease, but the changes did not reach statistical significance.

### Relationship Between Changes in BP and Body Weight After Administration of SGLT2 Inhibitors

We examined the relationship between the decrease in BP and body weight 6 months after administration of SGLT2 inhibitors. No significant association was found between the decreases in body weight and either SBP or DBP.

### Patients Who Could Stop or Reduce Antihypertensive Medications

**Fig. 6:**
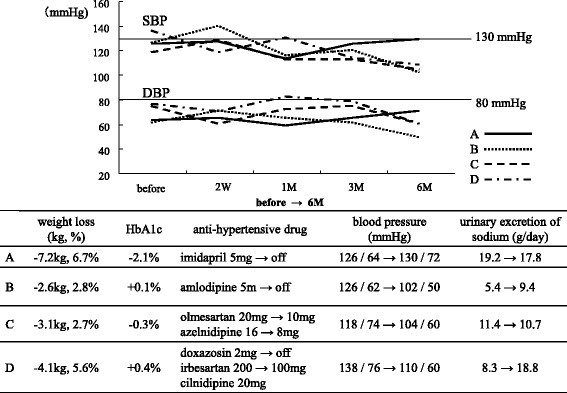
The clinical courses of four patients who could stop (A, B) or reduce (C, D) the amount of anti-hypertensive drugs. SBP, systolic blood pressure; DBP, diastolic blood pressure; HbA1C, glucosylated hemoglobin

Figure 6 demonstrates the clinical course of blood pressure in four patients who could mitigate the strength of their antihypertensive treatment 6 months after administration of SGLT2 inhibitors. Two patients could stop their use of antihypertensive agents; patient A decreased body weight by 7.2 kg and stopped taking imidapril 5 mg/day, and patient B lost 2.6 kg and discontinued amlodipine 5 mg/day. The other two patients could reduce the amount of antihypertensive agents; patient C reduced body weight by 3.1 kg and decreased the dose of olmesartan from 20 to 10 mg/day and azelnidipine from 16 to 8 mg/day, and patient D lost 4.1 kg and stopped taking doxazosin 2 mg/day and reduced the dose of irbesartan from 200 to 100 mg/day. Despite the reduction or withdrawal of antihypertensive medications, BP at 6 months was maintained below 130/80 mmHg in all four patients.

## Discussion

In the present study, both SBP and DBP as well as HbA1c level and body weight were significantly decreased after administration of SGLT2 inhibitors. The efficacy and mechanism of improvement of the HbA1c levels and weight loss by SGLT2 blocking are better understood [14, 15], but it remains unknown why inhibiting the SGLT2 channel decreases BP. To the best of our knowledge, this is the first report that investigated the mechanism of the BP-lowering effect of SGLT2 inhibitors using 24-h urinary collection and comparing the changes in urinary data with the decreases in BP at baseline and 2 weeks and 6 months after administration of the SGLT2 inhibitors.

In our study, there were significant correlations at both 2 weeks and 6 months after administration among the three measurements of urine volume, urinary glucose excretion, and urinary sodium excretion, except for the relationship between urine volume and urinary glucose excretion at 2 weeks that tended to be correlated but failed to reach statistical significance. SGLT2 is the major cotransporter involved in glucose reabsorption in the kidney [16]. SGLT transport links one glucose with one sodium ion for transportation into the proximal tubule cell. With inhibition of the SGLT2 protein, sodium reabsorption is reduced in the nephron, producing a mild diuretic effect [17, 18]. Increased glucose in the filtrate will maintain an increased urine volume through osmotic diuresis. Our results are consistent with the aforementioned mechanism of SGLT2 inhibitors.

SBP was significantly decreased at 1 month after administration of SGLT2 inhibitors. The decrease in SBP at 1 month was accompanied by an increase in urinary volume and urinary excretion of both glucose and sodium as well as body weight loss. The possible explanation is that plasma volume reduction caused by osmotic diuresis and natriuresis due to SGLT2 blocking may contribute to the decrease in SBP at 1 month. A previous study reported that administration of dapagliflozin, a kind of SGLT2 inhibitor, resulted in a 7% reduction in plasma volume estimated using a radioisotope technique with ^125^I-labeled human serum albumin at 4 weeks after administration [19]. In addition, in the present study, the decrease in SBP from baseline to 2 weeks after administration of SGLT2 inhibitors was significantly correlated with the increase in urinary glucose excretion during the same period. The key hemodynamic abnormalities related to SBP elevation are the increased cardiac output associated with the increased plasma volume [20]. A possible explanation of these findings is that in the relatively acute phase, the plasma volume reduction due to the increase in urinary glucose excretion induced by SGLT2 inhibition is the main contributor to the decrease in SBP.

SBP continued to decrease up to 6 months after administration of SGLT2 inhibitors. The decrease in SBP from baseline to 6 months was not correlated with the increase in urinary glucose excretion but was correlated with the increase in urinary sodium excretion, suggesting that the BP-lowering effect of SGLT2 inhibitors at 6 months could be explained mainly by plasma volume reduction due to natriuresis mediated by SGLT inhibition, not osmotic diuresis. DBP was significantly decreased for the first time at 6 months after administration of SGLT2 inhibitors. Body weight decreased significantly at 1 month and continued to decrease up to 6 months. A previous study also reported that the SGLT2 inhibitors induced clinically relevant and sustained weight loss through caloric loss due to the increased urinary glucose excretion. The fundamental hemodynamic fault associated with DBP elevation is an elevated systemic vascular resistance [20]. The vascular endothelial function is reported to be associated with obesity or the metabolic syndrome [21]. Vascular function, assessed by flow-mediated dilation, did not improve after 4 weeks of body weight reduction resulting from lifestyle changes [22], but improved significantly after 6 months of weight reduction [23]. It seems that the effects of body weight reduction on vascular function take some time to appear, which may explain the reduction of DBP by SGLT2 inhibitors at 6 months. Consequently, the BP-lowering effect at 6 months after administration of SGLT2 inhibitors is considered to be due to the plasma volume reduction resulting from the increase in urinary sodium excretion and improvement of vascular endothelial function related to body weight loss induced by SGLT2 inhibition.

In our study, SBP was significantly decreased in patients taking antihypertensive drugs. We think this is because 82% of patients prescribed an antihypertensive drug were taking an angiotensin converting enzyme inhibitor (ACEI) or angiotensin II receptor blocker (ARB) in the present study. The use of hydrochlorothiazide in addition to ARB was reported to show stronger antihypertensive effects compared with only ACEI or ARB [24] and the combination of ARB and diuretics is preferred in the treatment of hypertension [25]. Another study suggested that dapagliflozin potentiated the antihypertensive effects of calcium blockers and β-blockers in patients already receiving renin-angiotensin-aldosterone system (RAAS) blockers [26]. Because of its diuretic-like properties, the SGLT2 inhibitors combined with RAAS inhibitors might contribute to the SBP-lowering effects [19]. RAAS inhibitors were useful for preventing the development of diabetic kidney disease [27] and the guidelines of AHA/ACC and JSH recommend the use of RAAS inhibitors as a first-line drug for hypertensive patients complicated with diabetes [28, 29]. The effects and mechanisms of the combined use of RAAS inhibitors and SGLT2 inhibitors on plasma volume, renal function, and electrolytes should be further investigated.

SGLT2 inhibitors are antidiabetic agents that are irrespective of the patient’s insulin secretory capacity or insulin resistance and are clearly different from those of existing drugs. Furthermore, they have unique additional effects, including body weight loss and lowering BP. SGLT2 inhibitors may present a new treatment option for type 2 diabetes. In the EMPA-REG OUTCOME trial, the additional use of empagliflozin significantly improved cardiovascular outcomes in patients with type 2 diabetes at high cardiovascular risk [10] and significantly reduced onset or worsening of nephropathy in subgroup analysis [11]. In addition, a recent study has demonstrated that additional use of liraglutide, a glucagon-like peptide 1 analog, also improved cardiovascular outcomes in patients with type 2 diabetes at high risk [30]. The SGLT2 inhibitors and liraglutide have similar effects regarding the reductions in body weight and BP, which may play an important role in the prognostic improvement in patients with type 2 diabetes. Furthermore, to explain the early effects of improving cardiorenal outcome, a novel hypothesis has emerged, that the shifting of myocardial and renal fuel metabolism to ketone bodies promoted by SGLT2 blocking may contribute to cardiorenal work efficiency and function [31, 32]. Further studies are warranted to determine the precise mechanisms involved in improving cardiorenal outcome by administration of SGLT2 inhibitors.

Several limitations exist in our study. First, it was a single-center study with a relatively small number of patients because the 24-h urinary collection is bothersome to perform. According to the post hoc power analyses (α: 0.05, sample size: 20), the statistical power (1-β) of each analysis was as follows: urine volume vs sodium excretion at 2 weeks (R = 0.69), 0.97; glucose excretion vs sodium excretion at 2 weeks (R = 0.72), 0.99; urine volume vs sodium excretion at 6 months (R = 0.61), 0.91; urine volume vs glucose excretion at 6 months (R = 0.50), 0.76; glucose excretion vs sodium excretion at 6 months (R = 0.51), 0.77. Considering the minimal correlation coefficient in our study, at least 29 patients were necessary to ensure statistical power for all Pearson’s correlation analyses. A multicenter study with a large number of patients is necessary to validate the results of the present study. Second, our study lacked the comparison to a control group or analysis during the pre-administration period, which was necessary to clarify the effects of SGLT2 inhibitors on urinary measurements. Further studies including control groups should be performed in the future. Third, because of the study protocol dealing with several kinds of SGLT2 inhibitors, we could not determine the effects of any particular SGLT2 inhibitor in this study. We think the BP-lowering effect is a class effect of SGLT2 inhibitors. Finally, we lacked data regarding plasma renin activity and sympathetic nerve activity, which may contribute to the underlying mechanism for the BP-lowering effect of SGLT2 inhibitors.

## Conclusions

Although SGLT2 inhibitors may reduce BP through several mechanisms, our results suggest that plasma volume reduction, induced by osmotic diuresis at 2 weeks and by natriuresis at 6 months after administration of SGLT2 inhibitors, plays a key role in the BP-lowering effects. Further investigations are needed to attain a greater insight into these mechanisms necessary for understanding the potential cardiovascular benefit of SGLT2 inhibitors.

## Abbreviations

ACEI: Angiotensin converting enzyme inhibitor
ARB: Angiotensin II receptor blocker
BP: Blood pressure
DBP: Diastolic blood pressure
HbA1c: Glycosylated hemoglobin
RAAS: Renin-angiotensin-aldosterone system
SBP: Systolic blood pressure
SGLT2: Sodium-glucose cotransporter 2
